# The ‘Sessiz İstila’ dataset: emotion-annotated turkish tweets on refugee discourse and emoji lexicon

**DOI:** 10.1016/j.dib.2026.113284

**Published:** 2026-09-23

**Authors:** Hasri Akbar Awal Rozaq, Darmawan Wicaksono, Nevfel Boz, Mahmud Isnan, Bens Pardamean

**Affiliations:** a Bioinformatics and Data Science Research Center, Bina Nusantara University, Jakarta 11530, Indonesia; b Bureau of Communication and Public Relations, Ministry of Primary and Secondary Education, Jakarta 10270, Indonesia; c Department of New Media and Communications, Social Sciences University of Ankara (ASBU), Hükümet Meydanı No. 2, Ulus, Altındağ, 06050 Ankara, Türkiye; d Computer Science Department, School of Computer Science, Bina Nusantara University, Jakarta 11530, Indonesia; e Computer Science Department, BINUS Graduate Program – Master of Computer Science, Bina Nusantara University, Jakarta 11530, Indonesia

**Keywords:** Berturk, Emoji lexicon, Emotion recognition, Social media corpus, Turkish natural language processing

## Abstract

This article describes an emotion-annotated corpus of Turkish-language posts published on X (formerly Twitter) that discuss ‘sessiz istila’ (‘silent invasion’), a contested phrase used in Turkish public debate to frame the presence of refugees, particularly Syrian refugees, as a threat. We retrieved posts using the academictwitteR interface to the Twitter Academic Research API with the keyword ‘sessiz istila’, restricted to publicly available Turkish-language posts published between 1 June 2021 and 31 December 2022, yielding 47,024 posts. We normalized every post with a rule set implemented in R that replaces retweet markers, URLs, user mentions, and hashtags with Turkish placeholder tokens, converts text to lowercase, and substitutes each emoji with a Turkish description drawn from a purpose-built emoji lexicon of 3664 entries released alongside the corpus. Emotion labels were assigned automatically by an Emotion Recognition Model obtained by fine-tuning BERTurk (bert-base-turkish-cased) on a class-balanced subset of the TREMO dataset containing 18,018 validated entries, 3003 for each of the six Ekman categories (happiness, fear, sadness, disgust, surprise and anger). We retained predictions with softmax confidence below 0.6 but flagged them as ‘ambiguous’ rather than discarding them so that users can apply their own confidence policy. The released corpus therefore contains 40,880 confidently labeled posts and 6144 ambiguous posts, each with its creation timestamp, normalized text, date, year, and emotion label. The dataset supports secondary research on the circulation of anti-immigrant framing and on the reactions it provokes, on event-driven emotional dynamics in social media, and on benchmarking and error analysis of emotion classifiers for Turkish, an agglutinative and comparatively low-resource language. The balanced TREMO training file and the Turkish emoji lexicon are reusable across corpora.

Specifications Table

**Table:** 

Subject	Computer Science: Artificial Intelligence; Social Sciences: Communication
Specific subject area	Emotion recognition and natural language processing for Turkish social media discourse on refugees.
Type of data	Table; Text corpus; Lexicon. Processed (normalized text), Analyzed (model-assigned emotion labels). File format: CSV (UTF-8, comma-separated).
Data collection	We retrieved posts from X (formerly Twitter) using the academictwitteR R package via the Twitter Academic Research API on 26 January 2023, using the exact phrase ‘sessiz istila’ (exact_phrase = T) and limiting results to public Turkish-language posts published between 1 June 2021 and 31 December 2022; we obtained 47,024 posts. We normalized the text in R by replacing retweet markers, URLs, mentions, and hashtags with Turkish placeholder tokens; lowercasing characters; and replacing emojis with Turkish descriptions from an in-house lexicon. Duplicates were kept deliberately. Emotion labels were assigned by a BERTurk model fine-tuned on a class-balanced TREMO subset; labels below 0.6 softmax confidence are marked ambiguous.
Data source location	Data were generated by users of X and were collected and curated at: Social Sciences University of Ankara (ASBU), Ankara, Türkiye. Geographical scope of the discourse: Türkiye (Turkish-language posts). The data are stored and hosted at Zenodo (CERN, Geneva, Switzerland).
Data accessibility	Repository name: Zenodo Data identification number (DOI): 10.5281/zenodo.21897200 [1] Direct URL to data: https://zenodo.org/records/22804652 License: Creative Commons Attribution 4.0 International (CC BY 4.0) Instructions for accessing these data: The record is openly accessible without registration. Download the three CSV files from the record page and open them with any UTF-8-aware spreadsheet or statistical software (for example, pandas.read_csv with encoding='utf-8′).
Related research article	D. Wicaksono, H.A.A. Rozaq, N. Boz, Emotion recognition for low-resource Turkish: fine-tuning BERTurk on TREMO and testing on xenophobic political discourse (companion research article) [2].

## 1 Value of the Data

• The dataset provides the first openly available, emotion-annotated Turkish corpus devoted to the ‘sessiz istila’ (‘silent invasion’) framing of refugees. This phrase is central to anti-immigration rhetoric in Türkiye [3]. Existing accounts of this discourse rest on critical discourse analysis of party manifestos and on qualitative content analysis of video comments; these data instead couple 47,024 posts with a creation timestamp and a six-category emotion label, so that the same discourse can be examined quantitatively and longitudinally. For scale, TREMO, the reference emotion dataset for Turkish and the source of the fine-tuning material released here, comprises 27,350 entries elicited from 4709 respondents [4]; the corpus released here is about 1.7 times that size and consists of naturally occurring posts rather than elicited prose. The novelty claimed above is not a claim of size: it is that no emotion-annotated corpus of the ‘sessiz istila’ discourse was openly available before this release.
• The corpus was collected through the Twitter Academic Research API with the academictwitteR package [5] while that route was still open to researchers. Because free API access has since been withdrawn [6], the dataset preserves a time window (June 2021 to December 2022) that can no longer be reconstructed under comparable conditions, making it a reference corpus for replication and comparison with post-2023 material.
• Computational linguists working on Turkish, an agglutinative and comparatively low-resource language [7], can reuse the class-balanced training file derived from TREMO [4] (18,018 entries, 3003 per emotion) as a ready-made, bias-controlled fine-tuning set. This complements earlier Turkish emotion resources built as lexicons [8], word norms [9], and deep neural classifiers over tweets [10].
• The Turkish emoji lexicon (3664 entries mapping Unicode code points to Turkish descriptions [11]) fills a documented gap, since no comparable Turkish emoji description set was available. It can be reused independently for the pre-processing of any Turkish social media corpus, where the treatment of such tokens is known to affect transformer-based sentiment models [12].
• Low-confidence predictions are retained and flagged as ‘ambiguous’ (6144 posts, 13.1% of the corpus) instead of being deleted. Users can therefore reproduce the reported analyses at the 0.6 confidence threshold, exclude the ambiguous rows entirely, or study the ambiguous subset itself as material for research on annotation difficulty and on semantically overlapping emotions such as anger and fear. The probability distribution behind every label is released as softmax_scores_corpus.csv, so a user can impose any other threshold, or dispense with thresholding and work with the distribution itself, without running the model again.
• Because each record carries a timestamp and duplicates are retained, the data support work on emotionally driven diffusion, for which emotionally charged messages are known to be shared more than neutral ones [13] and can be aligned with external event chronologies such as the release of the short film Sessiz Istila on 3 May 2022, enabling event-study designs in computational social science, media studies, and migration studies. A concrete question the data answer in this form is whether the film shifted the emotional composition of the discourse or only its volume: the label distribution of posts published before 3 May 2022 can be compared directly with that of the posts published after it, against the 1451 posts collected in 2021 as a pre-event baseline, with the ambiguous rows included or excluded as a robustness check.

## 2 Background

The phrase ‘sessiz istila’ (‘silent invasion’) gained prominence in Turkish social media as a way of framing the presence of refugees, and especially Syrian refugees, as a cultural, economic, and demographic threat, and it circulates within a broader current of populist nationalism and anti-refugee sentiment in Türkiye [3]. Work on this discourse has so far been mainly qualitative, documenting its hostility through discourse and content analysis [14] but does not measure how the emotional composition of the discourse changes over time or at scale.

Measuring that composition computationally is difficult for Turkish. Turkish is agglutinative, so a single root can carry positive or negative meaning depending on its suffixes, and emotion resources developed for English transfer poorly, which is why dedicated Turkish resources have had to be built as word norms [9], emotion lexicons [8], and elicited labeled corpora such as TREMO [4]. Transformer models pre-trained on Turkish corpora, particularly BERTurk [15], an adaptation of the BERT architecture [16] to Turkish corpora, improve on these approaches [7,10]. The same pattern holds for other comparatively low-resource languages, where fine-tuned BERT variants outperform earlier approaches on affect-related classification [17]. Emoji resources are a concrete instance of the same gap: no Turkish emoji description set with coverage comparable to the English-language Unicode emoji list was available for pre-processing. However, the treatment of such tokens measurably affects transformer-based sentiment models [12].

This dataset was therefore compiled with two objectives: to make available a large, timestamped, and consistently normalized corpus of the ‘sessiz istila’ discourse, and to release the auxiliary resources needed to process it, namely a class-balanced Turkish emotion training file derived from TREMO [4], labeled with the six basic emotions [9] and a Turkish emoji lexicon [11]. In relation to the companion research article [2], which reports the architecture and evaluation of the Emotion Recognition Model, this data article adds the complete file-level description, column dictionary, distributional summaries, and processing provenance needed to reuse the data independently of that analysis.

## 3 Data Description

The dataset is deposited at Zenodo under DOI 10.5281/zenodo.21897200 [1] and consists of seven files placed at the root of the record: six comma-separated UTF-8 files and the fine-tuned model checkpoint that produced the emotion labels placed at the root of the record, with no folder hierarchy. Table 1 lists the files; Table 2, Table 3, Table 4 and Table 11 document the columns of the tabular ones. Tables 5, 6 and 8 summarise the distribution of the emotion labels in the annotated corpus; Table 7 reports the stance of a random sample of the distinct texts; and Table 9 illustrates the effect of the normalization rules. The code is openly available at https://github.com/dwicak/TwitterScrapping-for-Sezzis-Istila. The repository contains the R Markdown script that issues the retrieval call of Section 4.1 together with the exploratory frequency and network analyses, a second R Markdown script implementing the normalization rules and the emoji substitution of Section 4.2, and the Python notebook used to fine-tune the Emotion Recognition Model and to run the inference of Section 4.6 (Table 3).

**Table 1:** Files contained in the Zenodo record 10.5281/zenodo.21897200.

File name	Rows (excl. header)	Size	Content
silentinvasionv4_2021_2022_with_emotions_v14.csv	47,024	9.8 MB	Main corpus: normalized Turkish posts on ‘sessiz istila’ with timestamp and model-assigned emotion label
silentinvasionv4_2021_2022_with_emotions_v14_EN.csv	47,024	16.7 MB	English-translated counterpart of the main corpus: the same five columns plus text_EN, a machine translation of the normalized Turkish text
TREMODATAcalibrationApr2024.csv	18,018	1.2 MB	Class-balanced subset of the TREMO emotion dataset used to fine-tune the model (3003 entries per emotion)
TREMODATAcalibrationApr2024_EN.csv	18,018	2.1 MB	English-translated counterpart of the balanced TREMO subset: the same two columns plus Entry_EN, a machine translation of the Turkish entry
emojis_emoticons_Turkish.csv	3664	351.7 kB	Turkish emoji lexicon mapping Unicode code points and English descriptions to Turkish descriptions
softmax_scores_corpus.csv	47,024	4.4 MB	Per-post softmax probability distribution over the six emotion categories, row-aligned with the main corpus through created_at; lets users relabel the corpus at any confidence threshold (Table 11).
model_tremo_v13	—	442.6 MB	Fine-tuned BERTurk checkpoint that produced the released emotion labels, saved as a PyTorch state dictionary (Section 4.5).

**Table 2:** Column Dictionary of silentinvasionv4_2021_2022_with_emotions_v14.csv.

Column	Type	Description	Example
created_at	string (ISO 8601, UTC)	Timestamp of publication as returned by the API	2022–12–30T23:36:25.000Z
text	string	Normalised post text (lower-cased; placeholders substituted; emojis translated)	(retweetlemek) işid karargahını türkiyeye mi taşıdı? …
date	string (YYYY-MM-DD HH:MM:SS)	Publication time truncated to the minute, used for aggregation	2022–12–30 23:36:00
year	integer	Calendar year of publication (2021 or 2022)	2022
emotion	categorical (7 levels: the six emotion labels plus ‘ambiguous’)	Model-assigned label: anger, disgust, fear, happy, sadness, surprise, or ambiguous when the softmax confidence is below 0.6	sadness
text_EN	string	Machine translation of the normalized text into English (file silentinvasionv4_2021_2022_with_emotions_v14_EN.csv only); not human-verified	(retweet) did isis move its headquarters to turkey? …

Note: created_at, date and year are deliberately redundant. created_at is the ISO 8601 UTC timestamp returned by the API and is the authoritative time field, kept unaltered so that rows can be matched against the source platform and against other collections. date truncates it to the minute and year reduces it to the calendar year; both are derived and are supplied because they are read directly as a date and as an integer by the spreadsheet and statistical software commonly used for the aggregations reported in Section 3, whereas the trailing-Z form is frequently imported as text. Neither derived column carries information that cannot be recomputed from created_at, and users who prefer a single time field may drop them.

**Table 3:** Column Dictionary of TREMODATAcalibrationApr2024.csv.

Column	Type	Description	Example
Entry	string	Turkish sentence written by a respondent in the TREMO elicitation study	insanların düşünmeden hareket etmeleri.
ValidatedEmotion	categorical	Human-validated emotion label (anger, disgust, fear, happy, sadness, surprise); 3003 rows per label	anger
Entry_EN	string	Machine translation of the entry into English (file TREMODATAcalibrationApr2024_EN.csv only); not human-verified	People acting without thinking.

**Table 4:** Column Dictionary of emojis_emoticons_Turkish.csv.

Column	Type	Description	Example
Unicode.Code.Point.s.	string	Unicode code point(s) of the emoji; entries whose glyph is composed of several code points list them in order, separated by a single space	U + 1F600 (single code point); U + 1F1F9 U + 1F1F7 (sequence)
Emoji	string	The emoji character itself	
Description	string	Official English description	Grinning Face
Translated	string	Turkish description substituted into the text during normalization	Sırıtan Yüz

**Table 5:** Distribution of confidently labeled posts by emotion and year.

Emotion	2021–2022 count	2021–2022%	2021 count	2021%	2022 count	2022%
anger	17,839	43.6	303	20.9	17,536	44.5
surprise	13,656	33.4	815	56.2	12,841	32.6
fear	4581	11.2	135	9.3	4446	11.3
sadness	3512	8.6	185	12.7	3327	8.4
happy	954	2.3	12	0.8	942	2.4
disgust	338	0.8	1	0.1	337	0.9
Total (confident)	40,880	100.0	1451	100.0	39,429	100.0
ambiguous (excluded above)	6144	–	51	–	6093	–

**Table 6:** Distribution of confidently labeled posts by emotion at row level and at distinct-text level.

Emotion	Posts (rows)	% of rows	Unique texts	% of unique texts
anger	17,839	43.6	2315	30.2
surprise	13,656	33.4	1979	25.8
fear	4581	11.2	1832	23.9
sadness	3512	8.6	818	10.7
happy	954	2.3	539	7.0
disgust	338	0.8	176	2.3
Total (confident)	40,880	100.0	7659	100.0
ambiguous (excluded above)	6144	–	739	–

**Table 7:** Stance of a random sample of 400 distinct normalized posts toward the ‘Sessiz İstila’ framing.

Stance toward the ‘sessiz istila’ framing	Posts	%	95% CI
Endorses or amplifies the framing	225	56.2	51.4–61.0
Mentions it without evaluation	97	24.2	20.3–28.7
Argues against it or defends refugees	54	13.5	10.5–17.2
Indeterminate (truncated or fragmentary)	24	6.0	4.1–8.8
Total	400	100.0	–

Two of the seven files are English-language counterparts of the Turkish data files, added so that the corpus can be inspected and filtered by researchers who do not read Turkish. They reproduce their source file row for row and column for column and add a single column, text_EN and Entry_EN respectively, containing an English translation of the Turkish text column. The translations were produced automatically with a large language model instructed to preserve register, sarcasm and profanity without softening them, to leave emoji, hashtags and handles unchanged, and to render the normalization placeholders consistently as (retweet), (link) and (user). All 25,062 distinct Turkish strings across the two files were translated, so no cell of either English column is empty. These columns are machine-generated and were not verified by a human translator: the Turkish columns remain the authoritative text, the emotion labels apply to the Turkish text and not to its translation, and the English columns should be treated as a reading aid rather than as data for measurement.

Of the 47,024 posts in the main corpus, 40,880 (86.9%) received a confident emotion label, and 6144 (13.1%) were flagged as ambiguous. Table 5 reports the distribution of confident labels for the full observation window and separately for each year; percentages are computed over confidently labeled posts only, so they are unaffected by the choice of confidence threshold. Anger (43.6%) and surprise (33.4%) dominate the corpus overall, but the two years differ markedly: surprise leads in 2021 (56.2%), whereas anger leads in 2022 (44.5%).

Because retweets and repeated posts were retained (Section 4.2), the row counts in Table 5 are not counts of distinct messages. The 47,024 rows correspond to 8398 distinct normalized texts, so 38,626 rows (82.1%) repeat a text that appears elsewhere in the file; 38,885 rows (82.7%) begin with the retweet placeholder, and the single most frequent text occurs 6215 times. Among the 8139 rows that are not retweets, only 14.6% are repeats, so the duplication is almost entirely retweet-driven rather than a product of repeated original posting. This matters for interpretation: Table 6 recomputes the distribution of Table 5 over distinct texts, and the balance shifts markedly. Anger falls from 43.6% of rows to 30.2% of distinct texts. In comparison, fear rises from 11.2% to 23.9%, showing that the row-level dominance of anger is driven by a small number of heavily retweeted messages rather than by a correspondingly large number of distinct angry posts. Users studying the composition of the discourse should therefore work from the distinct-text figures, whereas users studying circulation and diffusion should work from the row-level figures; both are available in the released file because no de-duplication was applied.

Retention of retweets has a second consequence for the text itself. The API returns the body of a retweet truncated to its first 140 characters and terminated by an ellipsis. Since retweets make up 82.7% of the rows, 30,929 rows (65.8%) carry text that is cut off in this way; among distinct texts, the figure is 1132 of 8398 (13.5%). Truncated records are identifiable in the file because they end with the ellipsis character. This matters in two ways for reuse. The classifier saw only the visible portion of these posts, which is one plausible source of low-confidence predictions, and any measurement of post length, keyword presence, or lexical content computed over the file will be computed on partial text for most rows. Users who require complete message text should restrict their analysis to records that do not end in an ellipsis, and users of the English columns should note that a translation inherits the truncation of its source.

Matching the keyword does not by itself make a post hostile to refugees, and the corpus should not be read as a collection of xenophobic speech. To put a figure on this, we drew a random sample of 400 distinct normalized texts (seed 42). We coded each one for its stance toward the ‘sessiz istila’ framing under a four-category rubric: endorsing or amplifying the framing, mentioning it without evaluation, arguing against it or defending refugees, and indeterminate. Table 7 reports the results. A little over half the sample endorses or amplifies the framing, a quarter mentions it without evaluation, and roughly one post in seven argues against it. The coding was performed automatically by a large language model following the written rubric and has not yet been validated against human annotation, so these proportions are indicative rather than exact. They are nevertheless enough to establish the point that governs reuse: the corpus documents the discourse surrounding the phrase, including opposition to it, so a study of anti-refugee expression would need to filter the corpus rather than take it whole.

The corpus is strongly concentrated in time. Table 8 shows the monthly volume of confidently labeled posts: 2022–05 alone accounts for 51.9%, and 2022–06 for a further 21.9%, so these two months contain almost three-quarters of all labeled posts. The 2021 portion is comparatively sparse (1451 posts), with a single visible peak in 2021–07 (1203 posts).

**Table 8:** Monthly volume of confidently labeled posts.

Month	Confidently labeled posts	% of corpus
2021–06	6	0.0
2021–07	1203	2.9
2021–08	58	0.1
2021–09	3	0.0
2021–10	4	0.0
2021–11	3	0.0
2021–12	174	0.4
2022–01	12	0.0
2022–02	89	0.2
2022–03	138	0.3
2022–04	256	0.6
2022–05	21,228	51.9
2022–06	8955	21.9
2022–07	2578	6.3
2022–08	2097	5.1
2022–09	1957	4.8
2022–10	216	0.5
2022–11	833	2.0
2022–12	1070	2.6
Total	40,880	100.0

The May and June 2022 concentration coincides with the release of the short film Sessiz İstila on 3 May 2022, which circulated widely on X and gave the phrase renewed currency, and with a period of intensified public debate about refugee policy in Türkiye. We report this coincidence as context for the shape of the corpus rather than as a causal claim: the collection was keyword-driven, and the released records carry no information about what prompted any individual post.

Table 9 documents the effect of the normalization rules, showing four original posts next to their released form. The released corpus contains only the normalized column; we removed the original post text and all user-level metadata (user names, identifiers, follower counts, geolocation) during curation and do not redistribute them.

**Table 9:** Examples of original posts and their released normalized form.

Original post	Original Translated	Released normalized text	English Translation
RT @Zalim_Fira: Sessiz istila https://t.co/tB5KS6U94u, https://t.co/2mg9KzOQhu	RT @Zalim_Fira: silent invasion https://t.co/tB5KS6U94u, https://t.co/2mg9KzOQhu	(retweetlemek) sessiz istila (bağlantı adresi) (bağlantı adresi)	(retweet) silent invasion (link) (link)
RT @SedefKabas: Sessiz istila… Sarsıcı gerçek… Sinsi işgal… #sessizistila #suriyeliler #sığınmacısorunu	RT @SedefKabas: silent invasion… shocking truth… insidious occupation… #silentinvasion #syrians #refugee crisis	(retweetlemek) sessiz istila… sarsıcı gerçek… sinsi işgal… (sessizistila) (suriyeliler) (sığınmacısorunu)	(retweet) silent invasion… shocking truth… insidious occupation… (silentinvasion) (Syrians) (refugee crisis)
@KaracasuHande Çok yazık. Sessiz istila 5 gelirse, sonunda RTE olursa hiç şaşırmayacağım.	@KaracasuHande What a shame. If “Silent Invasion 5″ comes out and RTE ends up in power, I won’t be surprised at all.	(kullanıcı) çok yazık. sessiz istila 5 gelirse sonunda rte olursa hiç şaşırmayacağım.	(user) What a shame. If “Silent Invasion 5″ comes out and RTE ends up in power, I won’t be surprised at all.
@KaracasuHande baş tacısın sessiz istila	@KaracasuHande You're the crown jewel, silent invasion	(kullanıcı) (elleri çırpmak)(elleri çırpmak)(elleri çırpmak) baş tacısın sessiz istila	(user) (clap)(clap)(clap) You're the crown jewel, silent invasion

Note: tokens shown in parentheses in the two right-hand columns are placeholders inserted by the normalization rules of Section 4.2: (retweetlemek)/(retweet) for retweet markers, (bağlantı adresi)/(link) for URLs, (kullanıcı)/(user) for mentions, and Turkish emoji descriptions. They are not literal content of the original post. Hashtags also appear in parentheses because the rule strips the # symbol and keeps the token.

The same information is shown graphically in Fig. 1, Fig. 2, Fig. 3. Fig. 1 gives the overall frequency of each emotion label in the corpus, while Fig. 2, Fig. 3 disaggregate the labels by month for 2021 and 2022 respectively, making the July 2021 and the May–June 2022 concentrations directly visible.

**Fig. 1:**
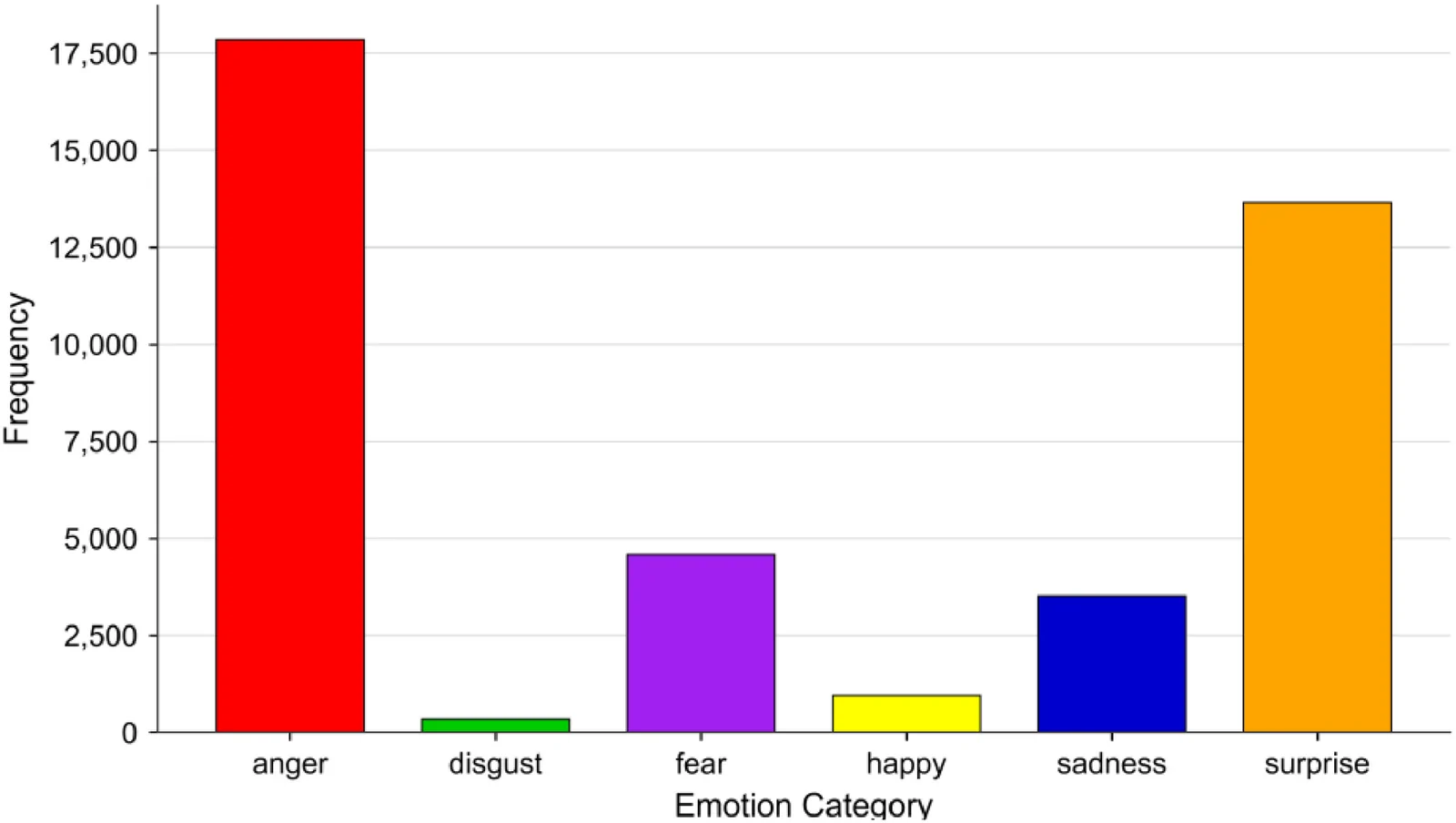
Frequency of each emotion label across the whole corpus (2021–2022).

**Fig. 2:**
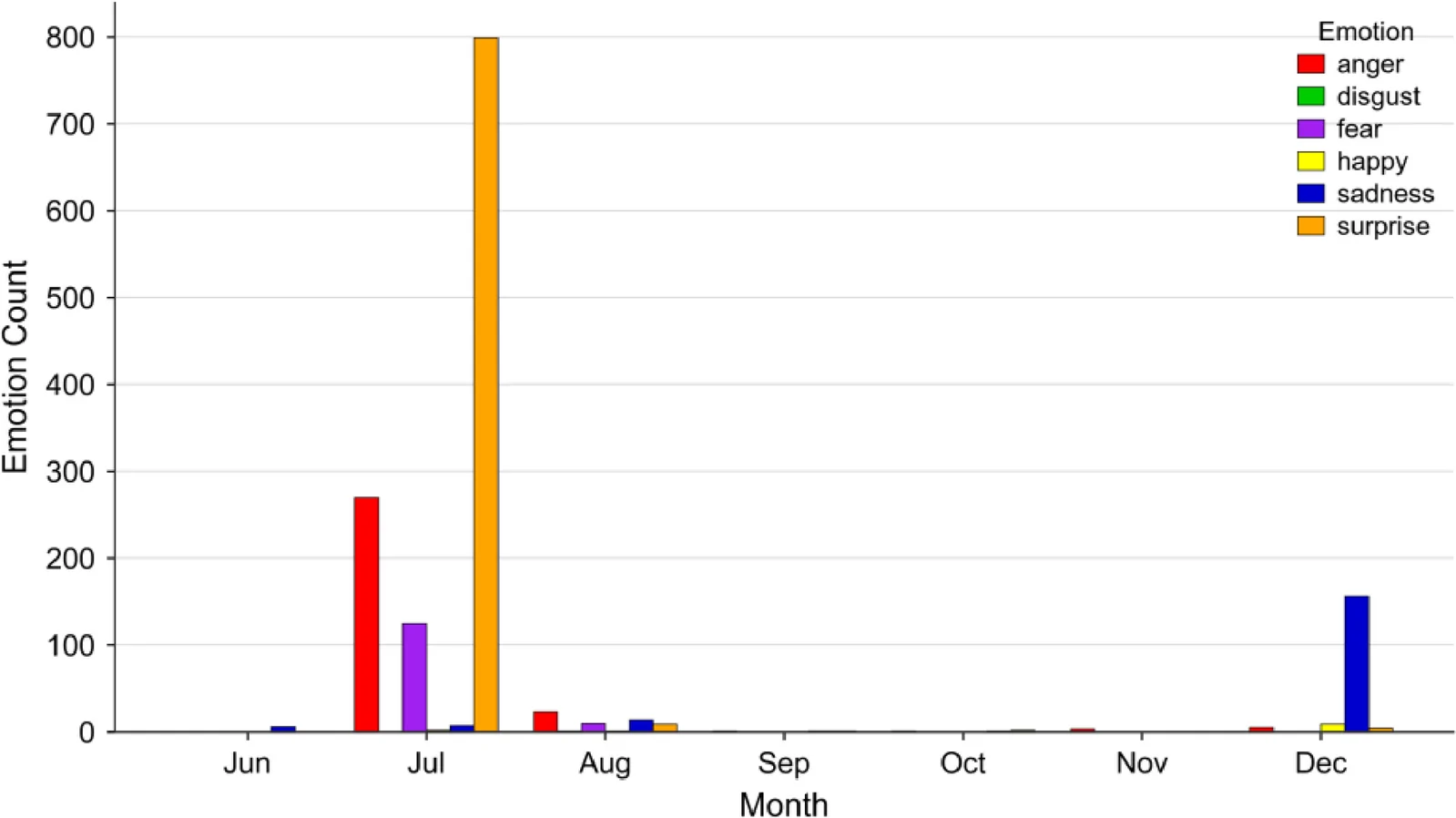
Monthly distribution of emotion labels in 2021.

**Fig. 3:**
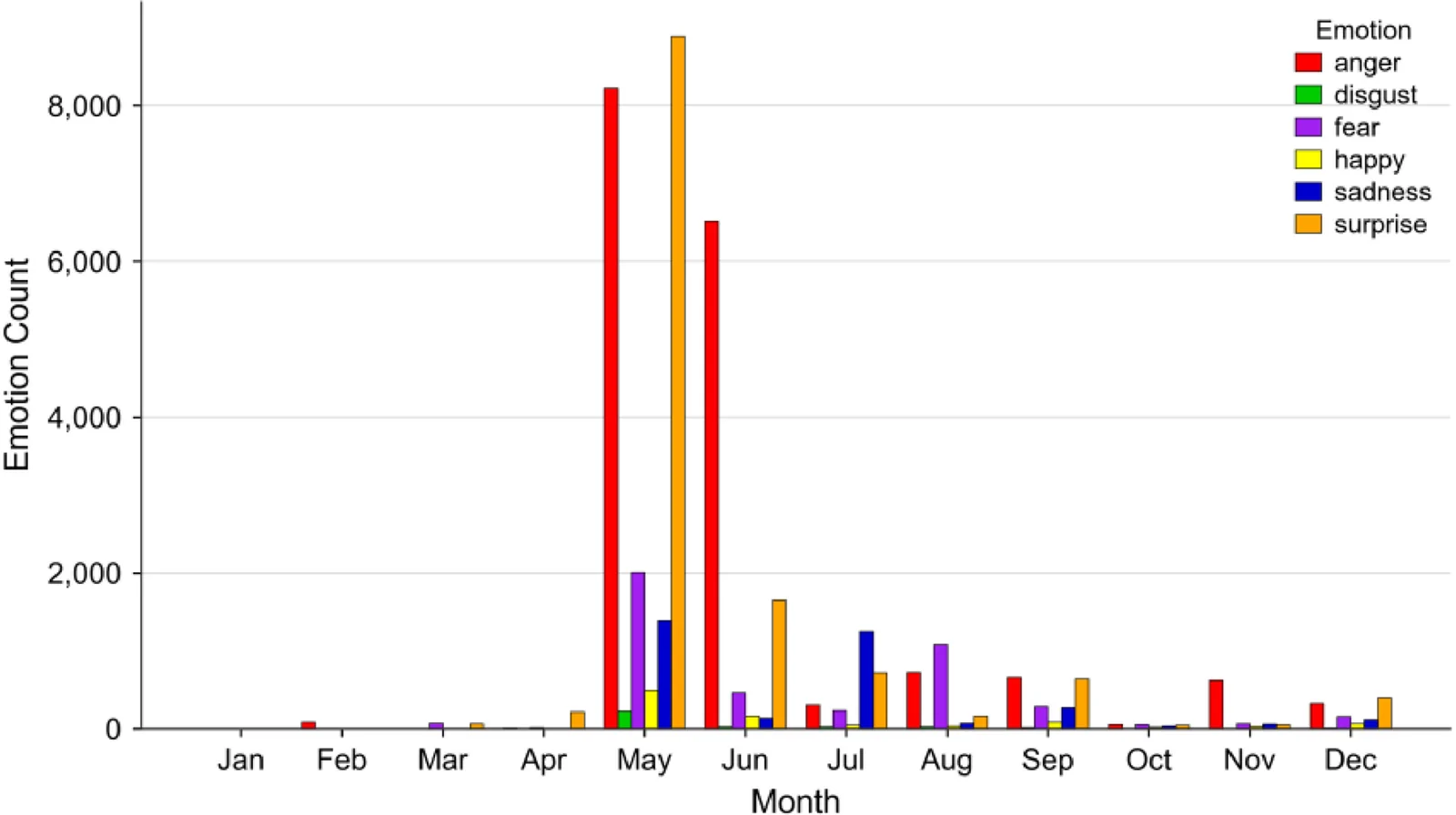
Monthly distribution of emotion labels in 2022,.

## 4 Experimental Design, Materials and Methods

Fig. 4 illustrates the workflow for constructing and evaluating the dataset. The following sections describe each process.

**Fig. 4:**
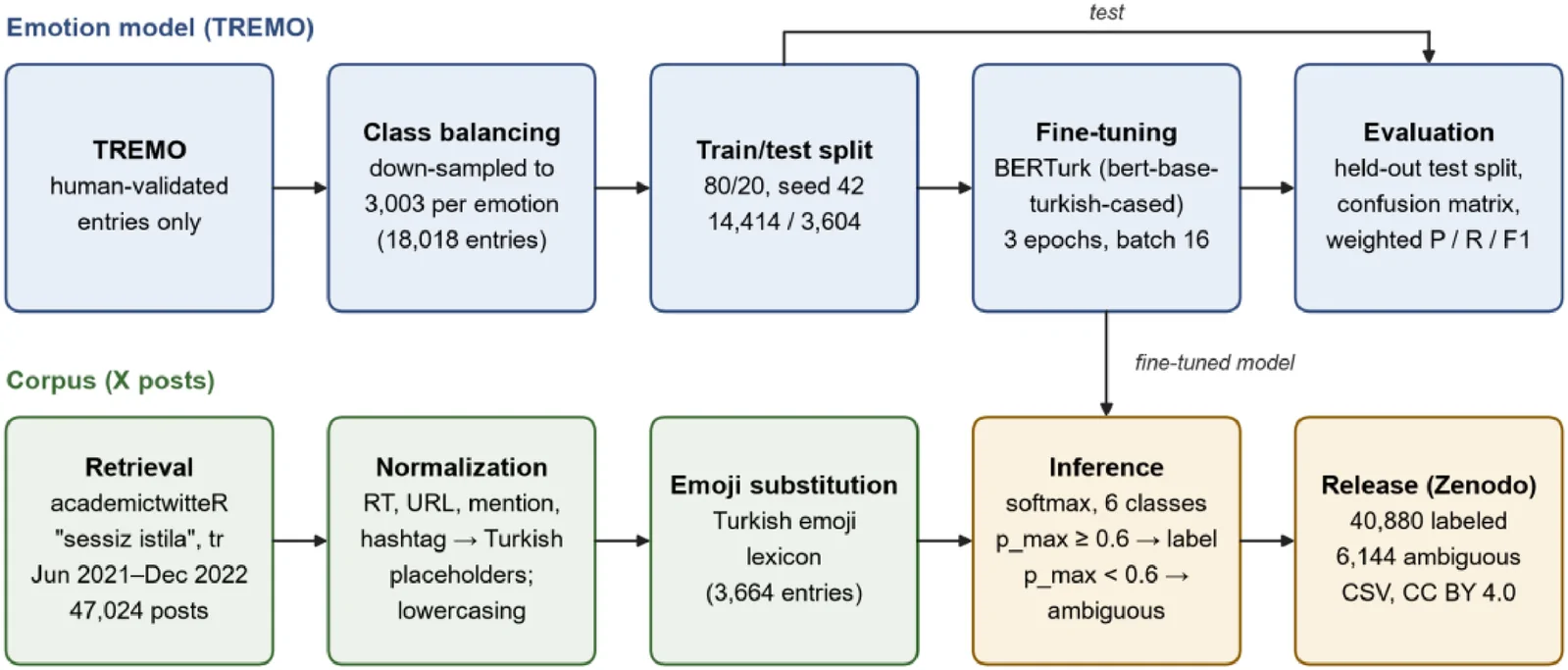
Workflow followed to collect, normalize, annotate, and release the data.

### 4.1 Data retrieval

Posts were retrieved from X (formerly Twitter) through the Twitter Academic Research product track using the academictwitteR package for R [5], executed on 26 January 2023 while free academic API access was still available. The retrieval call was, verbatim: get_all_tweets(query = "sessiz istila", exact_phrase = T, start_tweets = "2021–06–01T00:00:00Z", end_tweets = "2022–12–31T00:00:00Z", lang = "tr", n = Inf). Setting exact_phrase = T makes academictwitteR enclose the keyword in double quotes before submitting it to the v2 endpoint, so the two words were matched only as an adjacent phrase and never as a conjunction of the separate terms; matching is case-insensitive, and no further operators, filters or negations were used. The argument lang = "tr" restricted results to posts flagged by the platform as Turkish, and only posts publicly visible at retrieval time were returned; the window therefore runs from 1 June 2021 00:00 UTC to 00:00 UTC on 31 December 2022, which is why the latest record in the released file precedes that instant. Protected accounts, deleted posts, and non-Turkish results were therefore excluded by construction. The query returned 47,024 posts, and the earliest and latest records in the released file are 2021–06–08 10:00 and 2022–12–30 23:36, respectively.

### 4.2 Text normalization

Normalization was implemented in R with regular-expression substitutions applied in the following order, each replacing a platform-specific element with a Turkish placeholder so that the syntactic structure of the post is preserved while the identifying content is removed. The patterns are written as R string literals, in which the backslash is doubled, so \\w denotes the regular-expression token \w:

**Table:** 

"RT @\\w+:" → "(retweetlemek)" (retweet markers)
"\\w+://\\S+" → "(bağlantı adresi)" (URLs)
"@[a-z,_,A-Z,0–9]*" → "(kullanıcı)" (user mentions)
"#(\\w+)" → "(\\1)" (hashtags, hash symbol removed and the token kept)

Punctuation was deliberately kept because it carries information about sentence boundaries and emphasis that the transformer tokenizer can exploit. All characters were then converted to lowercase. Stop words were not removed and no stemming or lemmatization was applied, since pre-trained transformer models are known to lose accuracy when the input is stripped in this way. Duplicate posts were retained rather than de-duplicated, because repeated and retweeted content is itself the object of study in diffusion research on emotionally charged messages [13], and because pre-processing choices of this kind measurably affect transformer-based sentiment models [12].

### 4.3 Turkish emoji lexicon

No Turkish emoji resource with coverage comparable to the English-language Unicode emoji list was available, so we constructed one for this study by translating that list. Each entry in the released file emojis_emoticons_Turkish.csv [11] holds the Unicode code point, the emoji glyph, the official English description, and a Turkish translation of that description; the file contains 3664 entries. A Python routine scans every post for emoji glyphs and replaces them with the corresponding Turkish description in parentheses; for example,  becomes (elleri çırpmak). Emojis are therefore represented as ordinary Turkish tokens that the BERTurk tokenizer can process, rather than being discarded.

### 4.4 Training data preparation

Emotion labels were produced with a model fine-tuned on TREMO [4], a Turkish emotion dataset built from participant-written statements validated by human annotators. Only entries carrying a validated emotion were used. The validated pool is unbalanced, with surprise the smallest category at 3003 entries, so every category was down-sampled to that size, giving a balanced training file of 18,018 entries across the six Ekman categories [18]: happiness, fear, sadness, disgust, surprise, and anger, released as TREMODATAcalibrationApr2024.csv. We lowercased entries to match the corpus and encoded labels as happy = 0, fear = 1, sadness = 2, disgust = 3, surprise = 4, and anger = 5. We split the balanced file with scikit-learn train_test_split using test_size = 0.2 and random_state = 42, yielding 14,414 entries for training and 3604 entries for held-out testing. Fig. 5, Fig. 6 show the effect of this step: the validated TREMO pool is uneven across the six categories, whereas the released training file is uniform at 3003 entries per category.

**Fig. 5:**
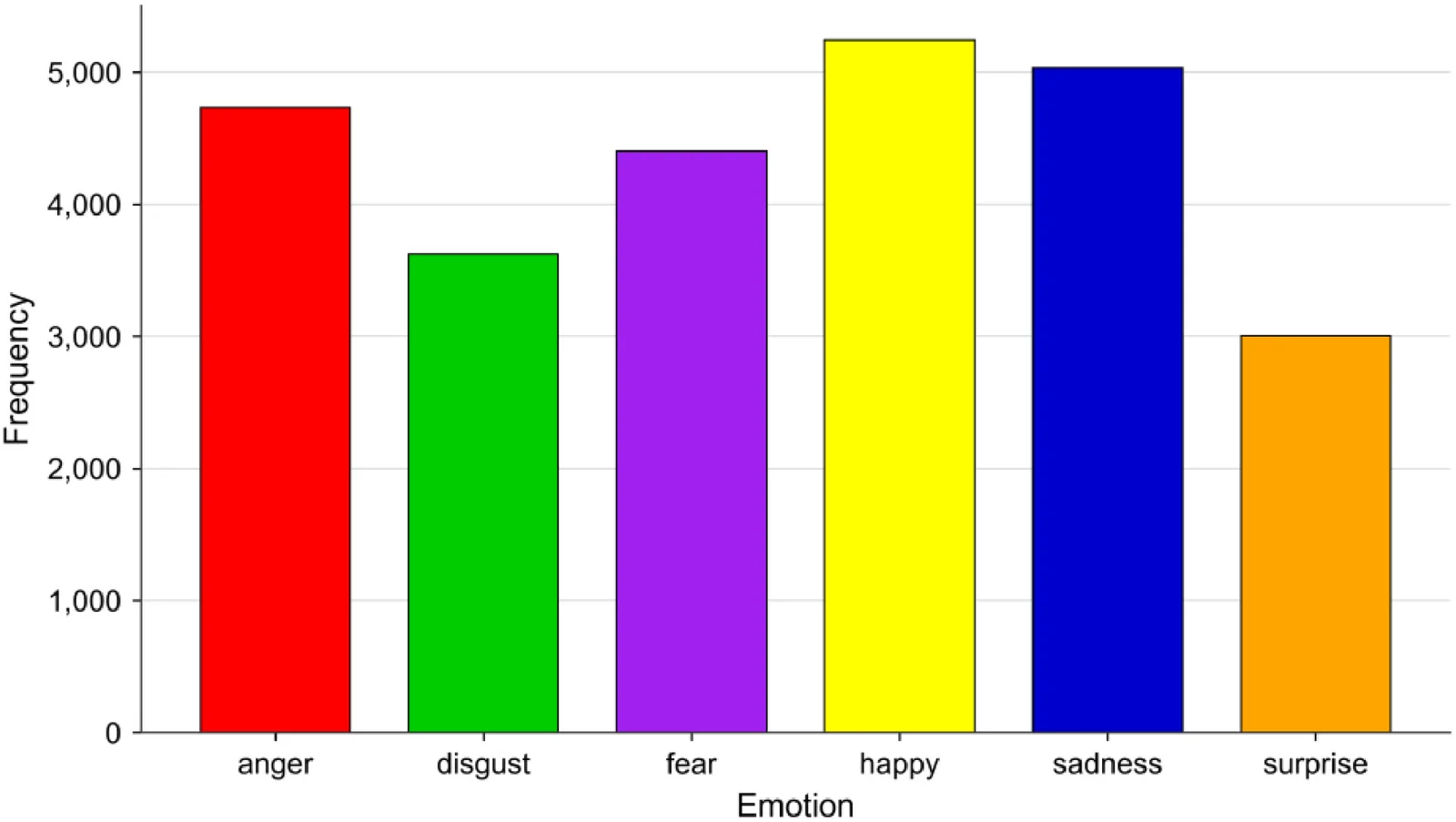
Distribution of the validated TREMO entries before balancing.

**Fig. 6:**
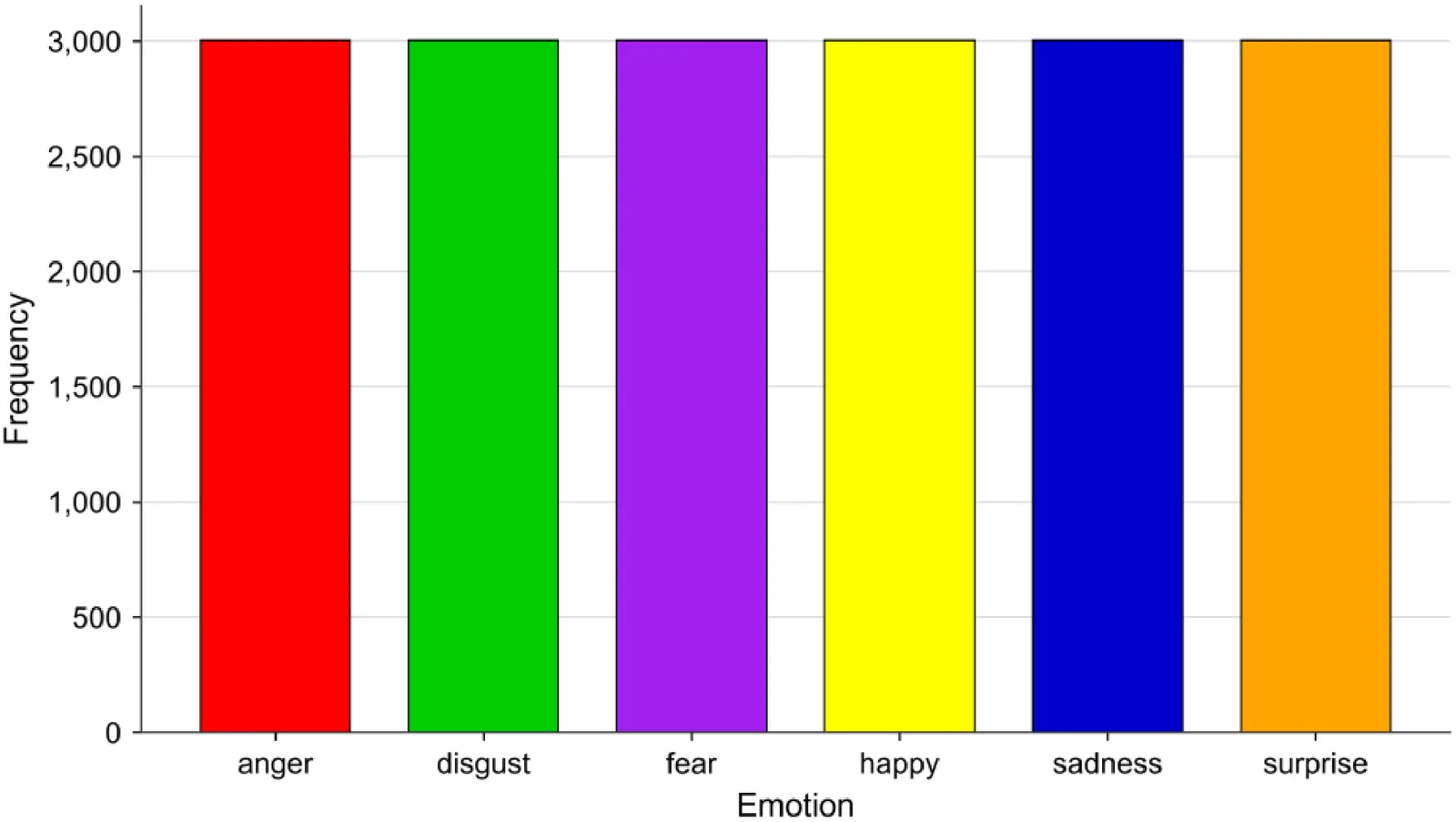
Distribution of the released training file TREMODATacalibrationApr2024.csv after balancing (3003 Entries Per Emotion).

The balanced file was then divided into a training and a test partition by a random 80/20 split (train_test_split, test_size = 0.2, random_state = 42), giving 14,414 training and 3604 test entries. The split was not stratified. Stratification was unnecessary here because the file is balanced by construction at exactly 3003 entries per emotion, so a simple random draw is expected to reproduce the uniform class proportions to within roughly one percentage point per class at this sample size; imposing stratification would have constrained the partition without materially changing its composition. The fixed seed makes the partition reproducible, and the released file contains all 18,018 entries, so users who prefer a stratified or cross-validated design can construct one themselves.

### 4.5 Model fine-tuning

We tokenized text with the BERTurk bert-base-turkish-cased tokenizer, a Turkish-language model pre-trained on Turkish corpora following the BERT architecture [16], with truncation enabled and dynamic padding to the longest sequence in each batch, and converted it to tensor batches. Truncation is unproblematic here because posts are short, unlike long-document settings where input length is itself a design constraint [19]. We fine-tuned it with the Hugging Face Transformers Trainer API on a GPU runtime in Google Colab. The run was configured for three epochs over the 14,414 training entries with a training batch size of 16, the configuration reported in the companion research article [2]. The notebook also retains a second, manually written training loop that runs for six epochs; it is an exploratory variant kept for reference and was not used to produce the released labels, which come from the Trainer configuration of Table 10. The loss values quoted below were logged during a re-run of the same configuration on the same split, bounded at 1200 optimization steps so that evaluation could be recorded at every logging interval; the accuracy and the per-category scores are computed from the confusion matrix of Fig. 7 under Eqs. (1)-(3). Table 10 lists the full configuration; arguments left at Hugging Face defaults, including the learning rate, optimizer, learning-rate schedule, and random seed, are listed with the default value in effect. Performance on the held-out 20% split reached an overall accuracy of 92.62% (3338 correct predictions out of 3604), with per-category precision between 0.9118 and 0.9567, recall between 0.8992 and 0.9447, and F1 between 0.9070 and 0.9480. The highest F1 scores were obtained for happy (0.9480) and disgust (0.9401), and the lowest for sadness (0.9070) and anger (0.9106). In that re-run, training and validation losses converged and stabilized after approximately 200 optimization steps, with no divergence between the two curves.

**Table 10:** Configuration used to fine-tune the emotion recognition model.

Setting	Value	Source
Pre-trained checkpoint	dbmdz/bert-base-turkish-cased (BERTurk)	set explicitly
Classification head	BertForSequenceClassification, num_labels = 6	set explicitly
Training length	num_train_epochs = 3 (production run); metrics logged from a re-run of the same configuration bounded at max_steps = 1200	set explicitly
Training batch size per device	16	set explicitly
Evaluation batch size per device	64	set explicitly
Warmup steps	500	set explicitly
Weight decay	0.01	set explicitly
Learning rate	5e-5	Hugging Face default
Optimizer	AdamW (β1 = 0.9, β2 = 0.999, ε = 1e-8)	Hugging Face default
Learning-rate schedule	linear decay after warmup	Hugging Face default
Random seed	42	Hugging Face default
Maximum gradient norm	1.0	Hugging Face default
Tokenization during fine-tuning	truncation enabled; dynamic padding to the longest sequence in the batch	set explicitly
Tokenization during inference	max_length = 128, padding to max_length, truncation enabled	set explicitly
Train/test split	train_test_split, test_size = 0.2, random_state = 42, not stratified (14,414 / 3604)	set explicitly
Evaluation metrics	accuracy, precision, recall and F1, weighted average (Eqs. (1)–(3))	set explicitly
Evaluation and checkpointing	evaluation every logging interval (steps); checkpoint saved each epoch; logging_steps = 10	set explicitly
Hardware	single GPU on a Google Colab runtime	set explicitly

**Fig. 7:**
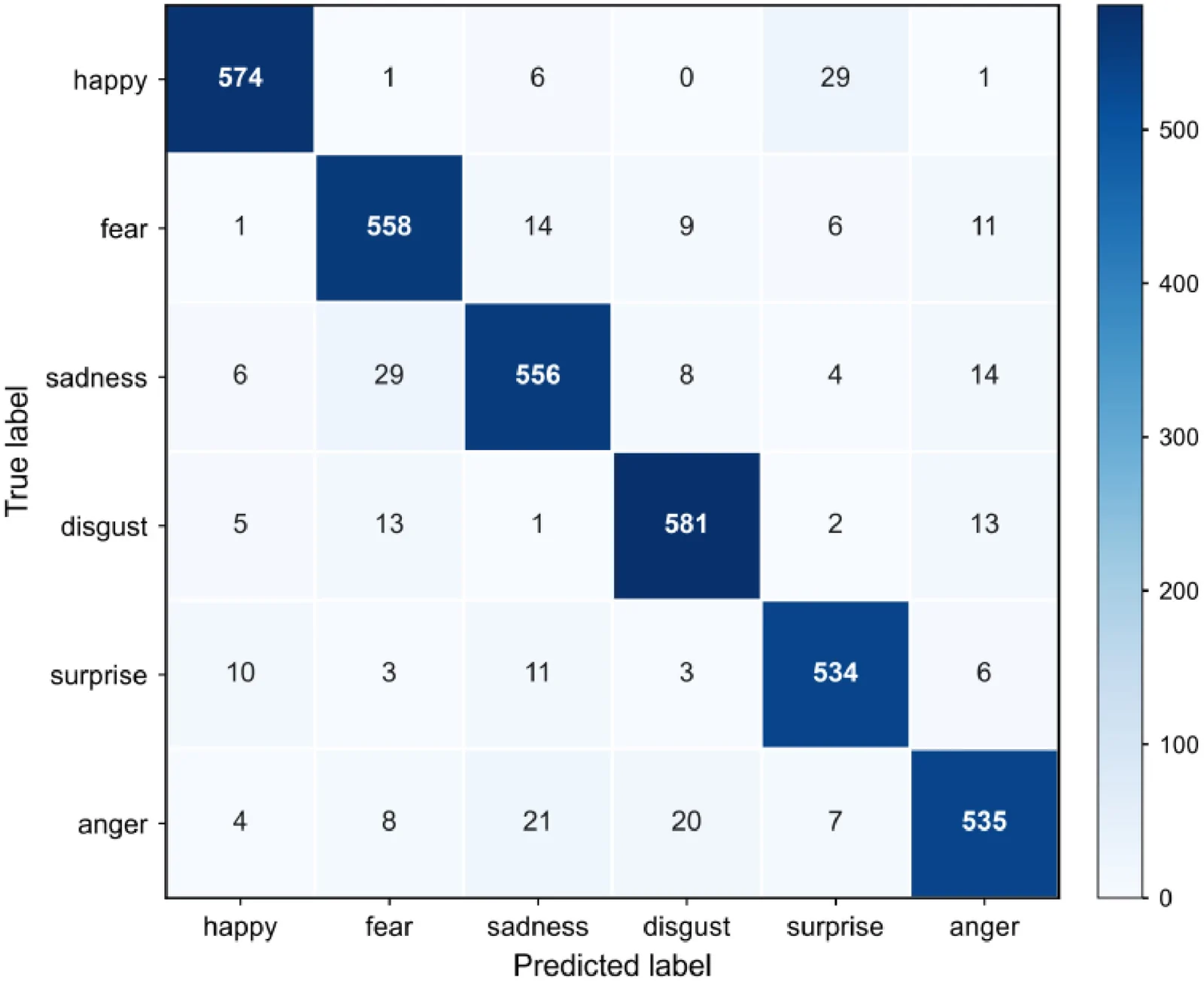
Confusion matrix of the fine-tuned model on the held-out 20% test split (3604 instances; 3338 correct predictions).

Precision, recall and F1 were computed for each of the six emotion categories i from the test-split confusion matrix, where TP_i_ is the number of test entries of category i predicted as i, FP_i_ the number of entries of other categories predicted as i, and FN_i_ the number of entries of category i predicted as another category:

(1) $$P_{i}=\frac{TP_{i}}{TP_{i}+FP_{i}},\;R_{i}=\frac{TP_{i}}{TP_{i}+FN_{i}}$$

(2) $$F1_{i}=\frac{2\cdot P_{i}\cdot R_{i}}{P_{i}+R_{i}}$$

The weighted averages listed in Table 10 weight each category by its support n_i_ = TP_i_ + FN_i_, the number of test entries that belong to it, so that the six supports sum to 3604:

(3) $$M_{w}=\frac{\sum_{i=1}^{6}n_{i}M_{i}}{\sum_{i=1}^{6}n_{i}},\;M\in \left\{P,\;R,\;F1\right\}$$

Weighted recall therefore reduces to overall accuracy, and because the test split was drawn from a class-balanced file the weighted averages differ from the unweighted means only in the fourth decimal place.

Fig. 7 gives the confusion matrix of the held-out test split, from which the accuracy of 92.62% and the per-category scores were computed. The optimization curves themselves, and the comparison of these figures with those reported by earlier Turkish emotion and sentiment classification studies, belong to the companion research article [2] and are not reproduced here. The numbers behind these figures are not deposited as separate supplementary files. The six-by-six count matrix lets a reader recompute accuracy and the per-category precision, recall and F1 by hand. The per-post softmax scores for the corpus are deposited in the record as softmax_scores_corpus.csv, together with the checkpoint that produced them (Section 4.6). The per-instance test predictions and the epoch-level training and validation loss values are not deposited separately; they remain reproducible, because the split is fixed by random_state = 42 under the configuration of Table 10.

### 4.6 Inference and labeling of the corpus

We applied the fine-tuned model to all 47,024 normalized posts. For each post, we tokenized the text with a fixed maximum length of 128 tokens and padded it to that length; we converted the raw logits to a probability distribution with softmax, and compared the highest probability against a confidence threshold of 0.6. Posts whose maximum probability met or exceeded the threshold received the corresponding emotion label; posts below the threshold were assigned the internal value −1, which is written to the released file as the string ‘ambiguous’. This procedure yielded 40,880 confidently labeled posts and 6144 ambiguous posts. We then exported the labeled records with the creation timestamp, a minute-resolution date field, and the calendar year, resulting in the five Turkish-language columns documented in Table 2. We retained both a full-precision timestamp and a minute-resolution field because created_at preserves the exact ordering of posts that diffusion analyses require. In contrast, the truncated date field is the key on which the daily and monthly aggregates reported in Section 3 were computed. We dropped all user-level metadata at this stage, so the published files contain no personal identifiers. The notebook writes this output under a working file name; the file released in the Zenodo record is silentinvasionv4_2021_2022_with_emotions_v14.csv, which is that output with the derived date and year columns added.

The threshold of 0.6 was not tuned against ground truth, because no human-annotated labels exist for these posts. Inference was run at 0.5, 0.6 and 0.7, and 0.6 was retained as the middle and more conservative of the three. It sits well above the 1/6 ≈ 0.167 that chance alone would give a six-way classifier and above the 0.5 point below which the leading class need not command a majority of the probability mass, while remaining low enough that most of the corpus still receives a label. At 0.6, 86.9% of posts were labeled. Because the value is a convention rather than an optimum, posts falling below it were flagged rather than deleted, so that users are free to include the ambiguous rows in their analyses or to exclude them, and Section 3 reports how much of the corpus each choice involves (40,880 labeled posts against 6144 ambiguous ones). Re-deriving labels under a different threshold requires the per-post softmax scores, which the record now contains.

The probability distribution behind every label is released as well. The file softmax_scores_corpus.csv holds one row per post, aligned with the main corpus through created_at, and reports the six class probabilities, their maximum, and the label that the 0.6 threshold yields; Table 11 documents its columns. The first version of the record did not contain this file, because the inference routine exported only the label that survived the threshold and discarded the distribution at the end of the run. Regenerating it faithfully required the exact checkpoint rather than a retrained equivalent, so that checkpoint is now deposited under the name model_tremo_v13, as a PyTorch state dictionary.

**Table 11:** Column dictionary of softmax_scores_corpus.csv.

Column	Type	Description	Example
created_at	string (ISO 8601, UTC)	Creation timestamp of the post, identical to created_at in the main corpus and the key that aligns the two files row by row	2022–12–30T23:36:25.000Z
prob_happy, prob_fear, prob_sadness, prob_disgust, prob_surprise, prob_anger	numeric (six columns)	Softmax probability assigned to each emotion category by the released checkpoint; the six values sum to 1 for every post	prob_sadness = 0.663160
max_prob	numeric	Largest of the six probabilities, that is, the value compared against the confidence threshold	0.663160
label_at_0.6	categorical (7 levels)	Label implied by the 0.6 threshold: the highest-probability category, or ‘ambiguous’ when max_prob is below 0.6. Identical to the emotion column of the main corpus on all 47,024 rows	sadness

A user who prefers a threshold other than 0.6 therefore needs no model at all: applying the chosen cut-off to max_prob in softmax_scores_corpus.csv relabels the corpus in a single pass, and the six probabilities also support analyses that dispense with a hard label. Regenerating the scores instead takes the notebook cited in Section 3: load model_tremo_v13 into a BertForSequenceClassification built on dbmdz/bert-base-turkish-cased with num_labels = 6, tokenize the text column of the corpus at max_length = 128 with padding to that length, and apply softmax to the logits, with the label ids of Section 4.4. Run in this way, the released scores reproduce the released emotion column on all 47,024 rows, yielding the same 40,880 labeled and 6144 ambiguous posts.

The emotion labels released in silentinvasionv4_2021_2022_with_emotions_v14.csv are model-generated and were not manually verified. Human validation enters this dataset only indirectly: the fine-tuning corpus is restricted to TREMO records with the ValidatedEmotion field, which the TREMO authors validated. The 92.62% accuracy reported above was measured on a held-out split of TREMO, a corpus of elicited emotion statements, and should therefore be read as an upper bound rather than as the accuracy attained on the X posts, which differ in register, length, and the presence of irony, slang, and emoji. The confidence threshold of 0.6 filters low-certainty predictions but does not verify correctness. Users who need verified labels for a subset of the corpus should manually re-annotate that subset.

### 4.7 Software

Collection and normalization were performed in R (version 4.6.1) with academictwitteR (version 0.3.1); emoji substitution, fine-tuning, and inference were performed in Python (version 3.13) with the Hugging Face Transformers (version 5.15.0) and PyTorch (version 2.13.0) libraries on Google Colab GPU runtimes; the balanced TREMO file, the corpus, and the lexicon were written as UTF-8 CSV. The R Markdown scripts and the Python notebook are deposited at https://github.com/dwicak/TwitterScrapping-for-Sezzis-Istila.

## Limitations

The emotion labels are model-generated rather than human-annotated, with a held-out accuracy of 92.62%, making them more appropriate as silver-standard labels. The six-category Ekman scheme also cannot capture emotions such as anxiety, hope, irony, and sarcasm. At the same time, using TREMO introduces a domain shift from elicited text to social media language.

The corpus is limited to posts containing “sessiz istila” and is therefore not representative of Turkish X users or refugee discourse. Nor is every post in it hostile to refugees: in a sample of 400 distinct texts, 56.2% endorse the framing while 13.5% argue against it (Table 7), so the corpus documents a contested debate rather than xenophobic speech alone. Most rows carry truncated text because the API returns retweet bodies cut to 140 characters, and 65.8% of rows end in an ellipsis. The data are temporally imbalanced, with 73.8% of labeled posts from May–June 2022. Retained duplicates make frequency reflect posting and retweeting activity rather than the number of distinct messages or authors: 82.1% of rows repeat a text that appears elsewhere in the file, almost entirely through retweets. The emotion distribution shifts appreciably when it is computed over distinct texts instead of rows (Table 6), so analyses of discourse composition should use the distinct-text figures. For privacy, we release only normalized text, with post IDs and user-level metadata removed.

## Ethics Statement

The authors have read and follow the ethical requirements for publication in Data in Brief. This work is based on data collected from a social media platform. We did not seek informed consent from individual account holders because the posts were publicly available at the time of retrieval; instead, we fully anonymized the participant data. During curation, we removed post identifiers, usernames, user identifiers, and all other user-level metadata; we then redistributed only the normalized post text, with user mentions replaced by a placeholder token, so the released files contain no personal identifiers, and no individual account can be traced. We collected the data through the Twitter Academic Research API and complied with the platform’s data redistribution policy [20]. The study involved no human participants and no animal experiments. The emotion labels released here were generated by the fine-tuned model rather than by human annotators; the human validation on which the model rests was carried out by the creators of the TREMO dataset [4], who validated the participant-written statements used for fine-tuning, and only entries carrying a validated emotion were used in this study.

## Credit Author Statement

**Hasri Akbar Awal Rozaq**: Software; Data curation; Validation; Visualization; Writing – original draft; Writing – review & editing. **Darmawan Wicaksono**: Conceptualization; Methodology; Software; Investigation; Data curation; Formal analysis; Writing – review & editing. **Nevfel Boz**: Conceptualization; Supervision; Validation; Writing – review & editing. **Mahmud Isnan**: Writing - Review & Editing. **Bens Pardamean**: Supervision.
